# Multicomponent Double-Hybrid Density Functional Theory

**DOI:** 10.1021/acs.jctc.5c01222

**Published:** 2025-11-07

**Authors:** Lukas Hasecke, Ricardo A. Mata

**Affiliations:** Institute of Physical Chemistry, University of Göttingen, Tammannstrasse 6, Göttingen 37077, Germany

## Abstract

In this contribution we investigate how far multicomponent density functional theory (DFT) results can be improved by the admixture of Møller–Plesset (MP) perturbation theory electron–proton correlation energies. Three formulations are explored, based off the popular double-hybrid functionals B2PLYP, DSD–PBEP86 and PBEQIDH. Partial use of the PA23 proton binding affinities data set is made to parametrize the ratio in the DFT/MP2 correlation energies. The resulting models are evaluated on a separate set of titratable molecules. The combination of nuclear electronic orbital (NEO) DFT and MP2 electron–proton correlation leads up to a 2-fold reduction in the root-mean-square deviation (RMSD) compared to standard NEO–DFT, a trend that is confirmed in the independent test set. We apply the parametrized NEO–B2PLYP model to compute the energetics of protonated water hexamers as well as a challenging example for proton dynamics, a crown ether molecule. In the latter case we compare the energetics of localized vs shared proton configurations. Overall, a ratio of about 0.8:0.2 (DFT/MP2) in the electron–proton correlation delivers a robust improvement across the models, even with variations in the basis sets used and the type of chemical bonds investigated.

## Graphical abstract

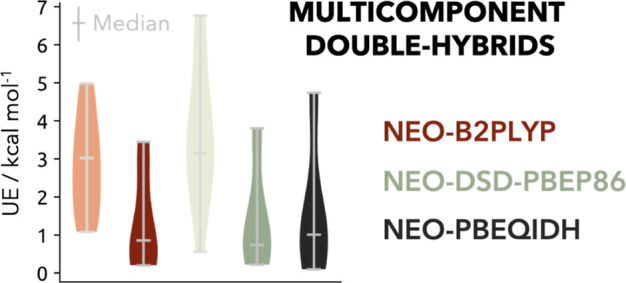

## Introduction

The description of proton dynamics is still today a challenging task. Due to the light mass of this particle, its wave-like (quantum) properties can be a factor even at room temperatures.^1,2^ The phenomenon of proton/hydrogen tunneling is well documented, being a common topic in both chemical and biochemical kinetics.^3–5^ Many hydrogen bonds can be involved in through-barrier tunneling, a highly correlated many-body problem.^6–9^ Also structures can be influenced by nuclear quantum effects (NQEs), for example in low-barrier hydrogen bonds which can significantly stabilize molecular aggregates or allow for structures which classically would not be allowed.^10–13^ The structure of water and its different phases are strongly influenced by NQEs as well.^14,15^ It is, therefore, not surprising that a plethora of methods to describe protons quantum mechanically has been proposed over the years.^16–20^

One particularly promising approach is that of multicomponent methods. In this framework, selected nuclei can be described on the same footing as electrons, with the total wave function for the system built as a combination of electronic and protonic wave functions. In the case of self-consistent field theories such as Hartree–Fock (HF) or DFT one works with the product of the two wave functions. The resulting algorithm is rather similar to an unrestricted electronic self-consistent field (SCF), with two Fock matrices (one for each subsystem) coupled through Coulomb interactions (in the case of HF) and electron–proton correlation when DFT is used. The exact functional for electron–proton correlation is, of course, unknown, and one needs to resort to approximations, usually inspired by the extensive body of work in electronic structure problems. To this date, the most commonly used electron–proton correlation functional is a local-density approximation (LDA) functional, the epc-17.2, put forward by Hammes-Schiffer and co-workers.^21^ It is based off the single-component formalism of the Colle–Salvetti electronic correlation energy. Other functionals have been suggested over time, most recently a functional by Holzer and Franzke for many-Fermion formalisms,^22^ whereby the correlation length for electron–proton can be straightforwardly adjusted.

Still, the development of (meta)­generalized gradient approximation (GGA) electron–proton correlation functionals remains challenging. One needs to work with both the electronic and protonic density gradients, which presents severe numerical problems. First of all, the maximum of the proton density tends to move away from the center of the atomic integration basins (the position of the classical proton), making it harder to accurately integrate over both the Fermionic gradients. This forces the use of large numerical integration grids, but even then SCF convergence might be problematic. Second, it is numerically more challenging to deal with the protonic gradients than with the electronic counterparts due to the larger range of values in the former.

In this work, we take a different approach to the electron–proton correlation. As noted above, many of the developments in multicomponent DFT take inspiration from single-component developments and theories. An approach that has been widespread for several years in electronic structure theory is the use of so-called double-hybrid functionals,^23,24^ which build on the idea of hybrid functionals such as the popular B3LYP, whereby HF exchange is admixed to the DFT energy. Double-hybrid functionals go one step further and take a combination of DFT and second-order Møller–Plesset perturbation theory (MP2) electronic correlation, the latter computed with Kohn–Sham orbitals. This has led to several functional forms with improved accuracy over their single-hybrid counterparts on a wide variety of benchmarks.^25–27^ We investigate how a similar combination could be used in the context of multicomponent DFT, admixing NEO–DFT and NEO–MP2 electron–proton correlation energies. We will refer to this approach as multicomponent double-hybrid functionals. We start by summarizing the basics of NEO–DFT. Later in the text we detail three formulations for the multicomponent double-hybrid functionals and the results of our benchmarks on how the DFT/MP2 admixture impacts the NEO results. Although not exclusively necessary, our formulations are combined with electronic double-hybrid DFT functional forms. We finally give some guidelines on the use of this methodology with a perspective on future work.

## Theory

### Multicomponent NEO–DFT

Multicomponent NEO–DFT presents a computationally low-cost alternative to capture nuclear quantum effects (NQEs) like anharmonic zero-point vibrational energies, delocalization and tunneling.^16,28–30^ It has been demonstrated that when these methods are paired with (local) density-fitting methodologies, large molecular sizes are amenable, with some of the largest calculations featuring tens of quantum protons.^31,32^ The total multicomponent energy can be computed from the one-particle densities of the electrons ρ^e^ and the protons ρ^p^, based on an adapted form of the Hohenberg–Kohn theorems.^33,34^ Moreover, by employing the multicomponent Kohn–Sham formalism the system is described as the product of the electronic and protonic Slater determinants built with the respective Kohn–Sham orbitals.^35,36^ Therefore, the total energy can be written as

1 $$E\left[\rho^{e},\rho^{p}\right]=E_{ext}\left[\rho^{e},\rho^{p}\right]+E_{ref}\left[\rho^{e},\rho^{p}\right]+E_{exc}\left[\rho^{e}\right]+E_{pxc}\left[\rho^{p}\right]+E_{epc}\left[\rho^{e},\rho^{p}\right]$$

where *E*_ext_[ρ^e^, ρ^p^] is the interaction of the respective densities with the external potential created by the classical nuclei, *E*_ref_[ρ^e^, ρ^p^] is the noninteracting kinetic energy and the Coulomb interaction between the quantum particles, *E*_exc_[ρ^e^] is the electron–electron exchange–correlation energy, *E*_pxc_[ρ^p^] is the proton–proton exchange–correlation energy and *E*_epc_[ρ^e^, ρ^p^] is the electron–proton correlation energy. Employing the variational principle results in a set of coupled Kohn–Sham equations with the effective potentials given as

2 $$\nu_{eff}^{p}\left(r_{1^{\prime }}\right)=\sum_{A}^{N_{c}}\frac{Z_{A}}{r_{1^{\prime }A}}-\sum_{i}^{N_{e}}\int \frac{|\phi_{i}^{e}\left(r_{1}\right){|}^{2}}{r_{11^{\prime }}}dr_{1}+\int \frac{\rho^{p}\left(r_{1^{\prime }}\right)}{r_{1^{\prime }2^{\prime }}}dr_{2^{\prime }}+K^{pp}\left(r_{1^{\prime }}\right)+\nu_{c}^{ep}\left(r_{1^{\prime }}\right)$$

3 $$\nu_{eff}^{e}\left(r_{1}\right)=-\sum_{A}^{N_{c}}\frac{Z_{A}}{r_{1A}}-\sum_{i^{\prime }}^{N_{p}}\int \frac{|\phi_{i^{\prime }}^{p}\left(r_{1^{\prime }}\right){|}^{2}}{r_{11^{\prime }}}dr_{1^{\prime }}+\int \frac{\rho^{e}\left(r_{2}\right)}{r_{12}}dr_{2}+\nu_{xc}^{ee}\left(r_{1}\right)+\nu_{c}^{ep}\left(r_{1}\right)$$

where the indices *A* indicate the *N*_c_ classical nuclei, *i* the *N*_e_ electrons and the dashed indices the *N*_p_ quantum protons. In case of the electronic effective potential ν_eff_^e^ two functional forms are required, the ν_xc_^ee^ electron–electron exchange–correlation functional and the ν_c_^ep^ electron–proton correlation functional. In order to obtain the electron–electron exchange–correlation energy any electronic functional can be employed within NEO–DFT, since it is defined identically to the conventional electronic functionals.^30^ The remaining electron–proton correlation can be approximated by different multicomponent correlation functionals which is not the focus of this study. For ease of discussion and interpretation we will consider only one electron–proton correlation functional, the epc-17.2.^21^ Regarding the protonic effective potential ν_eff_^p^, only the electron–proton correlation functional is included. Although it is possible to employ a specific proton–proton exchange–correlation function, the practical impact of these interactions for chemically relevant systems is negligible and leaving out this term reduces the computational costs of the NEO–DFT method. However, with the aim to eliminate self-interaction and since we do not use the independent proton approximation, the Hartree–Fock exchange *K* ^pp^ is included in the protonic effective potential.^37^

### Multicomponent Double-Hybrid DFT

Climbing up Jacob’s Ladder,^38^ double-hybrid density functionals that incorporate contributions from virtual Kohn–Sham orbitals provide a compelling approach to capture nonlocal correlation effects, especially important for long-range correlation effects like London-dispersion.^25^ The incorporation of virtual orbital contributions could be realized by various techniques such as Görling–Levy perturbation theory,^39^ optimized-effective-potential approaches,^40^ the random-phase approximation^41^ and wave function based correlation methods based on Kohn–Sham orbitals.^42^ First efforts in the direction of random-phase approximation within the context of multicomponent theory have already been made.^43^

We focus on the multicomponent extension of three commonly employed double-hybrid density functionals, namely B2PLYP,^24,44^ DSD–PBEP86,^45^ and PBEQIDH.^46^ These functionals exhibit diverse parametrization techniques for the admixture between DFT and Møller–Plesset second-order perturbation theory based correlation. This allows us to observe how the ideal ratio of DFT/MP2 electron–proton correlation depends (or not) on the other functional terms. In case of the NEO-B2PLYP method the total energy based on eq 1 is computed as

4 $$E_{NEO‐B2PLYP}\left[\rho^{e},\rho^{p}\right]=E_{ext,ref}\left[\rho^{e},\rho^{p}\right]+\left(1-a_{ex}\right)E_{ex}^{DFT}\left[\rho^{e}\right]+a_{ex}E_{ex}^{HF}\left[\phi^{e}\right]+\left(1-c_{ec}\right)E_{ec}^{DFT}\left[\rho^{e}\right]+c_{ec}E_{ec}^{MP2}\left[\phi^{e}\right]+b_{epc}E_{epc}^{DFT}\left[\rho^{e},\rho^{p}\right]+c_{epc}E_{epc}^{MP2}\left[\phi^{e},\phi^{p}\right]$$

where the parameters for the electronic exchange and correlation are set to *a*_ex_ = 0.53 for the amount of exact Hartree–Fock exchange and *c*_ec_ = 0.27 for the admixture of Møller–Plesset type correlation to the electron–electron correlation energy.^24^ These parameters were determined by a least-squares fit to the heats of formation within the G2/97 data set.^24^ The multicomponent Møller–Plesset second-order perturbation theory based electron–proton correlation energy *E*_epc_^MP2^ can be obtained with the electron–proton perturbation *W*_ep_ as^47^

5 $$E_{epc}^{MP2}\left[\phi^{e},\phi^{p}\right]=\sum_{\left(nn^{\prime }\right)\neq \left(00\right)}\frac{{\left|\left\langle \phi_{0}^{e}\phi_{0}^{p}\left|W_{ep}\right|\phi_{n}^{e}\phi_{n^{\prime }}^{p}\right\rangle \right|}^{2}}{E_{00}^{\left(0\right)}-E_{nn^{\prime }}^{\left(0\right)}}$$

where $|\phi_{n}^{e}\phi_{n^{\prime }}^{p}\rangle$ with (*nn*′) = (00) represents the zeroth-order wave function of the ground state for the electrons ϕ^e^ and protons ϕ^p^ and the excited states for (*nn*′) ≠ (00), and $E_{nn^{\prime }}^{\left(0\right)}$ the corresponding sum of the orbital energies. The parameters {*b*_epc_, *c*_epc_} for the admixture of epc-17.2 and Møller–Plesset type electron–proton correlation are determined within this work.

The NEO–DSD–PBEP86 version adds two further parameters for the spin-scaling {*c*_eco_, *c*_ecs_} of the opposite- and same-spin contributions to the electronic Møller–Plesset correlation energy. The total energy is given as

$$E_{NEO‐DSD‐PBEP86}\left[\rho^{e},\rho^{p}\right]=E_{ext,ref}\left[\rho^{e},\rho^{p}\right]+(1-c_{ex})E_{ex}^{DFT}\left[\rho^{e}\right]+c_{ex}E_{ex}^{HF}\left[\phi^{e}\right]+c_{ec}E_{ec}^{DFT}\left[\rho^{e}\right]+c_{eco}E_{eco}^{MP2}\left[\phi^{e}\right]+c_{ecs}E_{ecs}^{MP2}\left[\phi^{e}\right]+b_{epc}E_{epc}^{DFT}\left[\rho^{e},\rho^{p}\right]+c_{epc}E_{epc}^{MP2}\left[\phi^{e},\phi^{p}\right]$$ 6

with the parameters *c*_ex_ = 0.69, *c*_ec_ = 0.44, *c*_eco_ = 0.52, and *c*_ecs_ = 0.22. These are obtained by minimization of the arithmetic mean of the root-mean square deviations of six equally weighted data sets, covering the thermochemistry and kinetics of main group and transition metals as well as long-range interactions.^45,48^

The last double-hybrid functional chosen for this work is the NEO-PBEQIDH functional form. This functional differs from the previous two in the parametrization procedure, which is based on the adiabatic-connection formalism instead of an empirical parametrization to chemical reference data.^27,46,49^ The total energy of this functional is given as

7 $$E_{NEO‐PBEQIDH}\left[\rho^{e},\rho^{p}\right]=E_{ext,ref}\left[\rho^{e},\rho^{p}\right]+\left(1-\lambda_{ex}\right)E_{ex}^{DFT}\left[\rho^{e}\right]+\lambda_{ex}E_{ex}^{HF}\left[\phi^{e}\right]+\left(1-\lambda_{ec}\right)E_{ec}^{DFT}\left[\rho^{e}\right]+\lambda_{ec}E_{ec}^{MP2}\left[\phi^{e}\right]++b_{epc}E_{epc}^{DFT}\left[\rho^{e},\rho^{p}\right]+c_{epc}E_{epc}^{MP2}\left[\phi^{e},\phi^{p}\right]$$

where λ_ex_ = 3^–1/3^ and λ_ec_ = 1/3 are derived from first-principles utilizing a quadratic interpolation function to relate the real interacting system with the noninteracting particle system.^27,46,49^

## Computational Details

The functionals described above have been implemented in both their single- and multicomponent variants within the Molpro program package.^50^ A detailed description of our employed density-fitted NEO–MP2 and NEO–DFT implementations within Molpro can be found elsewhere.^32,50–52^

### Quantum Chemistry Calculations

All multicomponent NEO–DFT and NEO double-hybrid calculations reported in this work are conducted using the NEO module integrated in the developer version of the Molpro 2024.2 software package.^32,50–53^ In general, the PB4-F2 nuclear basis set together with the even tempered 10s10p10d10f density fitting basis set with exponents ranging from $2\sqrt{2}$ to 64 are employed for the quantum mechanical nuclei.^54^ Both NEO and single-component DFT calculations are carried out utilizing def2-TZVPP electronic basis sets with the def2-QZVPP-JKFIT density fitting basis set.^55,56^ For the subsequent MP2 calculation the def2-TZVPP electronic basis is employed together with the def2-QZVPP-MP2FIT density fitting basis set.^57^ Further orbital basis sets were also included to evaluate for basis set incompleteness effects, and how these may affect the parametrization. These include the PB6-F as nuclear basis^54^ but also the def2-QZVPP,^55,56^ aug-cc-pVTZ-mc,^58^ uncHq-def2-QZVPP^59^ and uncHq-aug-cc-pVTZ-mc^59^ electronic basis sets. Thereby, in case of the (uncHq-)­aug-cc-pVTZ-mc basis sets a union of the aug-cc-pVQZ-JKFIT (DFT) or aug-cc-pVQZ-MP2FIT (MP2) density fitting basis set with the even tempered 10s10p10d10f density fitting basis set is employed.^60,61^ For the (uncHq-)­def2-QZVPP electronic orbital basis set the respective def2-QZVPP-JKFIT (DFT) and def2-QZVPP-MP2FIT (MP2) density fitting basis sets are employed together with the even tempered 10s10p10d10f protonic density fitting basis set. A threshold of 10^–8^ Hartree was used for the energy difference within the electronic, nuclear SCF subcycles and the overall energy difference in the NEO–DFT iterations. For the difference in the density between iterations and the gradient of the respective nuclear and electronic subiterations a threshold of 10^–5^ a.u. is employed.^50^ All Molpro computations employ the direct inversion in the iterative subspace initiated after the first iteration with a total of 10 Fock matrices as basis to extrapolate.^62,63^ Throughout this work the standard grid 3 is employed for the computations.^64^ The electron–proton correlation is computed with the epc-17.2 functional.^21^ All calculations include the D3 dispersion correction with Becke–Johnson damping.^44,65^ Nuclear densities shown are computed at a 0.02 σ contour level generated with the PyMOL 2.5.2 program suite.^66^ The employed molecular systems are optimized with Gaussian16 employing the def2-TZVPP basis set, unless otherwise stated, with very tight SCF settings, tight optimization thresholds and a superfine grid.^67^ Subsequent frequency calculations are carried out with the same settings.

### Bayesian Optimization Settings

With the aim to find the optimal values for the admixture of electron–proton correlation obtained with the epc-17.2 functional (*b*_epc_) and from Møller–Plesset second-order perturbation theory (*c*_epc_), we employed a Bayesian optimization procedure. This machine learning method facilitates the efficient exploration of high dimensional parameter spaces to find the global minimum of a black-box target function.^68,69^ Within our approach the objective is to minimize the RMSD between the experimental reference values and those derived from the various NEO double-hybrid functionals. The Bayesian optimization employs Gaussian process regression, utilizing a Matérn52 kernel, to model the underlying function space. Additionally, the expected improvement acquisition function guides the selection of new parameters {*b*_epc_, *c*_epc_}, which are likely to yield improvements in the objective function, minimizing the overall RMSD. Thereby, the parameter space is restricted between 0 and 1 (100%). In an alternative approach close to the B2PLYP parametrization we optimize only the *b*_epc_ parameter and define *c*_epc_ = 1 – *b*_epc_.^24^ To ensure a comprehensive exploration of the parameter space and minimize uncertainty, we initiated the optimization process by sampling 40 points (10 points for the single parameter optimization) using Latin hypercube sampling.^70^ Subsequently, the next 20 points (3 points for the single parameter optimization) are based on the acquisition function. This iterative refinement on top of the presampling enables the optimization algorithm to focus on promising regions of the parameter space, ultimately converging toward optimal admixture factor of electron–proton correlation effectively and efficiently. The GPyOpt implementation is employed for the Bayesian optimization.^71^

### Reference Data Set

In order to provide a well established and ideal reference data set for multicomponent methods, the PA23 set of proton binding affinities developed by Hammes-Schiffer and co-workers is employed.^21^ This set contains 11 carboxylates, six amines, three aromatic systems, and three inorganic molecules, providing a chemically diverse data set for (experimental) proton affinities. The entire data set is displayed in Table 1. However, recently Moncada et al. highlighted the necessity of rotational corrections to restore the correct rotational symmetry of multicomponent wave functions in case of diatomic and nonlinear triatomic molecules.^72^ Moreover, the HCN molecule was employed for the parametrization of the epc-17.2 functional which could lead to a bias toward the DFT based electron–proton correlation within the multicomponent double-hybrid parametrization.^21^ Therefore, as a precaution we constrain the molecular space of the reference data set to the first 21 molecules, henceforth denoted as the PA21 data set. In general, the proton affinity (PA) describes the process of adding a proton to a molecule A

$$A+H^{+}\to {AH}^{+}$$

**Table 1:** Chemical Composition and Experimental Proton Affinities in kcal mol^–1^ of the PA23 Data set^a^

index	molecule	exp. PA
carboxylates		
**1**	HCOO^–^	345.2
**2**	CH_3_COO^–^	348.5
**3**	CH_3_CH_2_COO^–^	347.5
**4**	CH_3_(CH_2_)_2_COO^–^	346.6
**5**	CH_3_(CH_2_)_3_COO^–^	346.1
**6**	CH_3_COCOO^–^	333.5
**7**	CH_2_FCOO^–^	339.2
**8**	CHF_2_COO^–^	330.2
**9**	CF_3_COO^–^	322.6
**10**	ClCH_2_COO^–^	336.2
**11**	Cl(CH_2_)_2_COO^–^	340.8
amines		
**12**	NH_3_	204.1
**13**	CH_3_NH_2_	214.9
**14**	CH_3_CH_2_NH_2_	217.9
**15**	CH_3_(CH_2_)_2_NH_2_	219.3
**16**	(CH_3_)_2_NH	222.1
**17**	(CH_3_)_3_N	226.9
aromatics		
**18**	C_6_H_5_NH_2_	211.0
**19**	C_6_H_5_O^–^	351.4
**20**	C_6_H_5_COO^–^	340.1
inorganics		
**21**	NO_2_^–^	340.1
**22**	CN^–^	353.1
**23**	HS^–^	353.1

a The anions **22** and **23** are not considered in this work (PA21).^21^

The energy involved in this process can be approximated as the negative reaction enthalpy Δ*H* based on the reaction energy Δ*E* as

8 PA(A)=−ΔH=−ΔE+RT

whereby *T* is the temperature and *R* the universal gas constant. The total energy is composed of the temperature dependent kinetic energy of the different degrees of freedom, which are the translation, rotation and vibration, together with the electronic energy

9 $$E\left(T\right)=E_{trans}\left(T\right)+E_{rot}\left(T\right)+E_{vib}\left(T\right)+E_{elec}$$

Within the ideal gas approximation the translational energy change for the protonation is given as

10 $$\Delta E_{trans}\left(T\right)=-\frac{3}{2}RT$$

whereas the change of the rotational energy can be neglected due to the lacking rotational energy of the free proton and approximately same rotational energy of the protonated and unprotonated molecule A.^21^ Therefore, the proton affinity can be expressed as

11 $$PA\left(A\right)=-\Delta E_{elec}-\Delta E_{vib}+\frac{5}{2}RT$$

One advantage of multicomponent methods like NEO–DFT is that the anharmonic zero-point vibrational energy is inherently and accurately included for the quantum protons.^16,32^ Moreover, Pavošević et al. demonstrated that the zero-point vibrational energies of the classical nuclei associated with the protonated and unprotonated molecule predominantly cancel.^73^ Therefore, the proton affinity can be computed as

12 $$PA\left(A\right)=E_{A}-E_{{AH}^{+}}+\frac{5}{2}RT$$

where *E*_A_ is the electronic energy of a regular single-component based calculation for the unprotonated molecule A and *E*_AH^+^_ is the electronic energy of the protonated molecule obtained from a multicomponent method directly including the anharmonic zero-point vibrational energy of the quantum proton.

## Results and Discussion

We start by benchmarking the performance of conventional single-component hybrid and double-hybrid density functionals for the prediction of the experimental proton affinities within the PA21 data set. The overall unsigned errors (UE) as well as the individual errors for the respective molecules are shown in Figure 1 for the hybrid functionals B3LYP and PBE0 as well as for the double-hybrid functionals B2PLYP, DSD–PBEP86 and PBEQIDH. In regards to the median of the UE the best performing method is the double-hybrid B2PLYP with a median UE of 1.23 kcal mol^–1^ followed by the hybrid B3LYP counterpart with a slightly higher median of 1.80 kcal mol^–1^. The PBE based functionals exhibit a higher median compared to the latter ones. Best performing is the spin-scaled DSD–PBEP86 double-hybrid with a median of 1.95 kcal mol^–1^, followed by the hybrid PBE0 functional at 2.44 kcal mol^–1^. The double-hybrid PBEQIDH has the highest median UE of 3.16 kcal mol^–1^.

**Figure 1:**
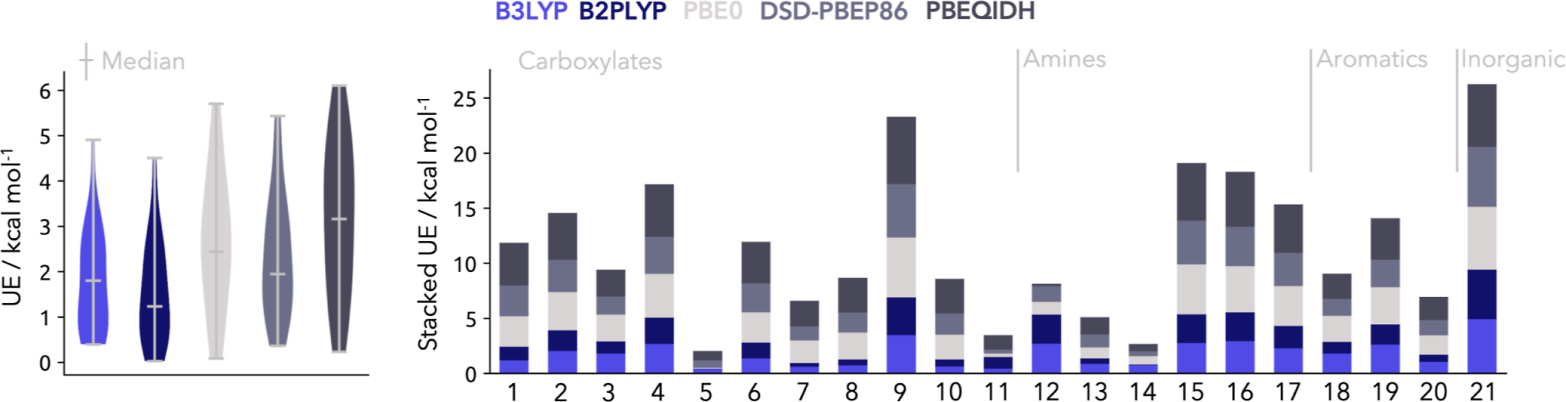
Left: Violin plots with highlighted median of the overall performance of the respective methods given as unsigned error on the PA21 data set. Right: Stacked unsigned errors of the respective methods for each molecule in the PA21 data set.

Analyzing the overall data distribution of the UEs it becomes apparent that B3LYP, B2PLYP and DSD–PBEP86 result in an uneven distribution exhibiting few outliers to higher UEs than the median and a compact distribution toward lower UEs below the median. On the other hand, PBE0 and PBEQIDH exhibit a rather uniform distribution of the UEs. Considering the lowest UEs, every functional shows values below the chemical accuracy of 1 kcal mol^–1^. The molecule with the lowest UE for B3LYP, B2PLYP and PBE0 is CH_3_(CH_2_)_3_COO^–^ (**5**) with respective UEs of 0.40, 0.03, and 0.09 kcal mol^–1^, respectively. In the case of DSD–PBEP86 the lowest UE is 0.36 kcal mol^–1^ for the Cl­(CH_2_)_2_COO^–^ (**11**) molecule, while PBEQIDH performs best for NH_3_ (**12**) with a UE of 0.22 kcal mol^–1^. The latter one is the only example of the chosen test functionals which performs better on amines, whereas the others best perform on carboxylates. However, over the whole data set there are quite some differences within each class of systems. While **5** is a positive example for the performance of carboxylates, the CF_3_COO^–^ (**9**) molecule exhibits large deviations for all functionals.

In fact, this molecule shows the largest UE determined by the PBEQIDH functional with 6.10 kcal mol^–1^. For all other functionals the highest UE is found for the NO_2_^–^ (**21**) molecule with UEs of 4.91, 4.51,5.70, and 5.43 kcal mol^–1^ for B3LYP, B2PLYP, PBE0, and DSD–PBEP86 respectively.

Overall, it becomes apparent that the chemical diversity of this data set is also reflected in the obtained UEs for the different functionals where no parametrization can describe the test systems equally well within or across different chemical motifs. Since this data set is already extensively used to benchmark multicomponent methods we would like to compare to some of the provided references.

Brorsen et al. archived a mean unsigned error (MUE) of 1.38 kcal mol^–1^ by employing NEO–B3LYP with the epc-17.2 functional utilizing a def2-QZVPP electronic basis set together with an even tempered 10s10p10d protonic basis set, also highlighting the impact of electron–proton correlation since leaving this contribution out increased the MUE to 17.98 kcal mol^–1^.^21^ Mejía-Rodríguez and de la Lande obtained a root-mean square deviation (RMSD) of 1.92 kcal mol^–1^ with their density-fitted NEO-B3LYP version utilizing the epc-17.2 functional with a def2-TZVPP electronic basis set, even tempered HET-5S5P3D nuclear basis set together with the GEN-A4* electronic auxiliary basis set and ET-8* auxiliary nuclear basis set.^31^ Recently, Khan and Tonner-Zech highlighted the significance of electron–proton correlation and a sufficient electronic basis set size for accurate proton affinities by employing multicomponent DFT, whereas the choice of the nuclear basis set only shows minor impact.^74^ Regarding the multicomponent density fitted wave function based performance on the PA23 set, the works of Pavošević et al. and Fetherolf et al. obtained a MUE of 7.38 kcal mol^–1^ for NEO–MP2 and 1.38 kcal mol^–1^ for spin-scaled NEO–MP2, utilizing the aug-cc-pVQZ electronic basis set paired with the aug-cc-pVQZ-RI electronic auxiliary basis set and the PB4-F2 nuclear basis set.^75,76^ For the spin-scaled NEO–MP2 results the employed electronic basis set on the classical nuclei is the aug-cc-pVTZ basis set.^76^ Overall, the results of NEO–DFT and NEO–MP2 outperform their single-component counterparts with the exception of the B2PLYP functional which is slightly more accurate on the PA21 test set. However, regarding hybrid functionals Kahn and Tonner-Zech demonstrated on a larger data set of 72 small organic molecules that NEO–DFT systematically produces more accurate results than single-component methods if electron–proton correlation is included properly.^74^ The question is whether the DFT/MP2 electron–proton correlation admixture can in fact systematically improve this description.

### Parametrization of Multicomponent Double-Hybrid Functionals

Following the expressions of eqs 4, 6, and 7 we will keep the electronic parameters untouched, as they are already fitted for the single-component counterpart. Only the fraction of correlation stemming from the epc-17.2 functional and NEO–MP2 will be tuned. This will be valid as long as the electronic density is only slightly impacted going from a single- to a multicomponent calculation. To validate this approach, we carefully analyzed the difference in the electronic reference density for both calculations. The obtained integrated electronic difference densities are shown for C_6_H_5_NH_3_, (CH_3_)_3_NH, and CF_3_COOH in Figure 2. It becomes apparent, that by describing the quantum nucleus as charge density the surrounding electronic density also becomes further delocalized. This issue is rather well-known, and the reason why electronic basis sets in use for NEO calculations are under discussion/revision.^58,77^ All of the analyzed systems show the same qualitative and quantitative trend of the difference density. The overall impact of the nuclear delocalization on the electronic density is relatively small. The largest difference is, as expected, at the position of the quantum hydrogen. The negative value indicates that more electronic density is accumulated on the point charge (H nucleus) in the single-component calculation. The multicomponent density is more delocalized, shifting toward the bond with the nitrogen or oxygen atom. However, a smaller fraction of the electronic density is also oriented further away from the bond. Moreover, even the surrounding of the bonding nitrogen and oxygen atoms displays a minor difference in the electronic density. One can observe a similar symmetric pattern as discussed before, electronic density is shifted further away from the classical nuclei. Clearly, this results from the increased electron density on the bond axis between the quantum proton and the classical nuclei. Overall, the difference in the electronic density within the single- and multicomponent pictures shows a robust trend across diverse chemical systems, has a minor quantitative impact, and is well localized around the quantum nucleus. The parametrization of the important electron–proton correlation via {*b*_epc_, *c*_epc_} heavily depends on the interplay of protonic and electronic density around the quantum nucleus. Errors from the original electronic parametrization in this region can be effectively compensated. Nonetheless, a full dimensional reparameterization would be desirable in the future, especially in the light of newly developed multicomponent basis sets and electron–proton correlation functionals. In the context of this study, we will restrict ourselves to the most commonly used EPC functional and nuclear orbital basis sets.

**Figure 2:**
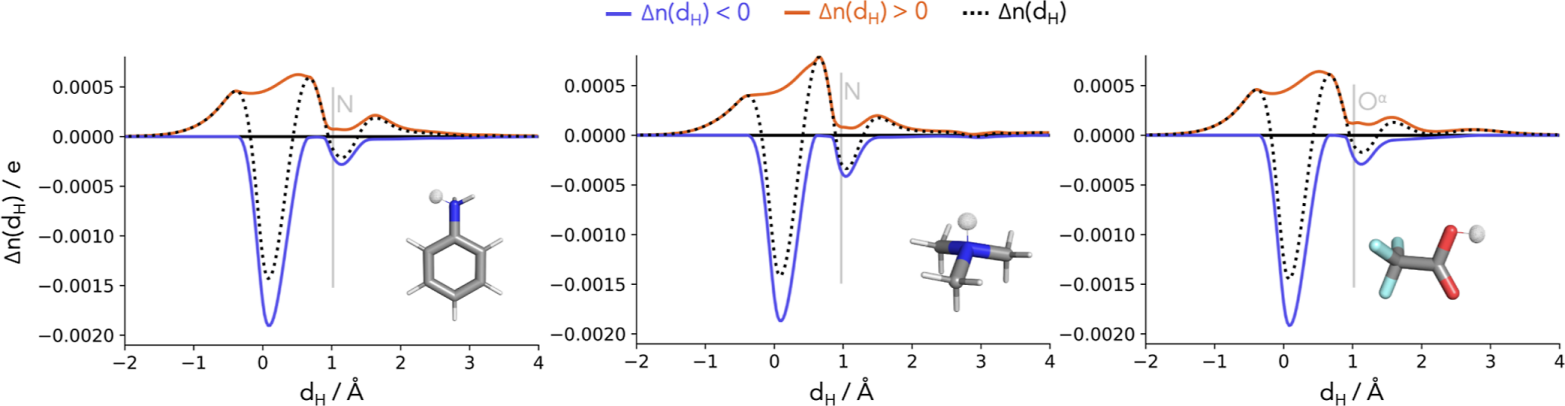
Integrated density differences Δρ = ρ_NEO_^e^ – ρ_SC_^e^ between the single-component (B3LYP) and multicomponent (NEO–B3LYP with epc-17.2) electronic densities for C_6_H_5_NH_3_ (left), (CH_3_)_3_NH (middle), and CF_3_COOH (right) along the bond-axis. Thereby, *d*_H_ is defined as the distance from the quantum proton position (*x* = 0) along the bond-axis with the classical nuclei (nitrogen or oxygen). The blue line highlights electronic depletion, corresponding to a larger electron count within the single-component method, the orange line indicates electronic excess, where the multicomponent electron count is larger. The black dashed line symbols the overall difference in the integrated density. The voxel dimensions utilized are 0.05 × 0.05 × 0.05 b.

The results of the Bayesian optimizations for the admixture of the DFT/MP2 electron–proton correlation on the PA21 reference data set of proton affinities are shown in Figure 3.

**Figure 3:**
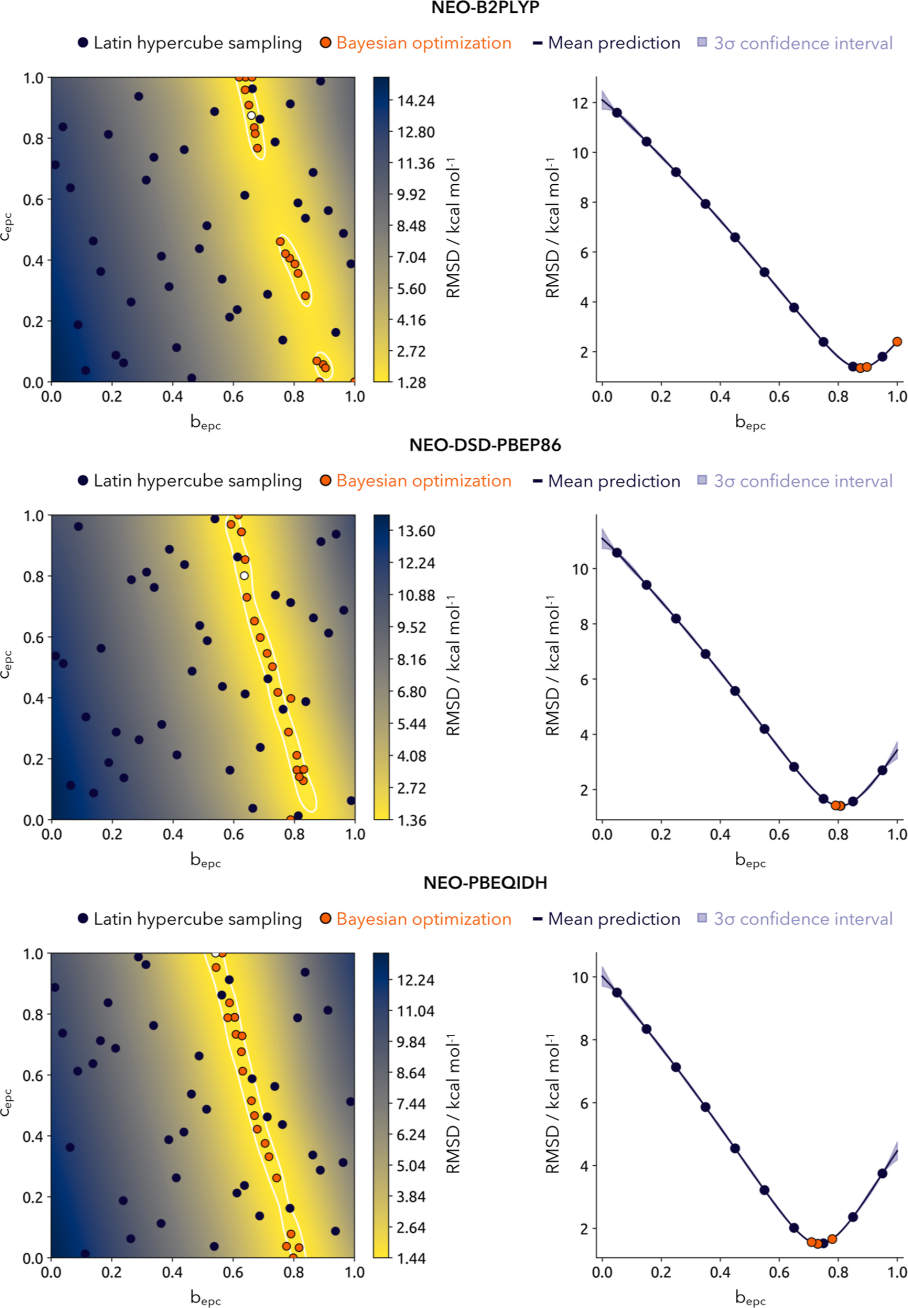
Bayesian optimization of the parameters for the admixture of electron–proton correlation obtained with the epc-17.2 *b*_epc_ and MP2 *c*_epc_ on the PA21 data set of proton affinities shown with the achieved RMSD for the respective double-hybrid density functionals. The left side shows the full two-dimensional optimization, where {*b*_epc_, *c*_epc_} are chosen independently and the right side is displaying the one-dimensional optimization of *b*_epc_, where *c*_epc_ = 1 – *b*_epc_. The white contour line in the two-dimensional surface highlights the area within 0.1 kcal mol^–1^ of the lowest obtained RMSD indicated as white point.

We start our discussion of the obtained results with the two-dimensional optimization of the parameter space {*b*_epc_, *c*_epc_}. For all chosen double-hybrid density functionals the same trend can be observed. While changes in the RMSD are strongly influenced by variations in the epc-17.2 *b*_epc_ parameter, the MP2-derived electron–proton correlation has a comparatively minor impact. All parameter surfaces show the same qualitative trend, forming a narrow valley along *b*_epc_ = 0.6–0.8. The difference in the RMSDs along the valley is rather small, slightly above 1 kcal mol^–1^, and is also very similar across all functionals. However, the valley itself becomes increasingly flatter from NEO–B2PLYP, to NEO–DSD-PBEP86, to NEO–PBEQIDH. The parametrization changes slightly across the three functionals. While NEO–B2PLYP has the optimal performance for higher correlation coming from the epc-17.2, the *b*_epc_ parameter is gradually decreasing for NEO–DSD–PBEP86 and NEO–PBEQIDH. Regarding the one-dimensional optimization (taking the diagonal, only values with *b*_epc_ + *c*_epc_ = 1), this trends becomes even more apparent. The slopes and widths of the valleys across the *b*_epc_ parameter are similar, with the optimal value found at around *b*_epc_ = 0.8. The obtained optimized parameters for both schemes are found in Table 2.

**Table 2:** Obtained Parameters for the Admixture of epc-17.2 (*b*_epc_) and MP2 (*c*_epc_) Electron–Proton Correlation Based on the One and Two Dimensional Bayesian Optimization with Resulting RMSDs in kcal mol^–1^

functional	*b*_epc_	*c*_epc_	RMSD
1-parameter optimization			
NEO–B2PLYP	0.875	0.125	1.34
NEO–DSD–PBEP86	0.806	0.194	1.41
NEO–PBEQIDH	0.731	0.269	1.51
2-parameter optimization			
NEO–B2PLYP	0.660	0.875	1.34
NEO–DSD–PBEP86	0.635	0.801	1.41
NEO–PBEQIDH	0.542	1.000	1.50

Although the obtained parameters for the admixture between the one and two-dimensional are somewhat different, the overall accuracy is similar. Given this fact, we opt for the 1-parameter optimization, as it is sounder from a physical perspective. Overall our results seem to indicate that an admixture of 0.8:0.2 is fairly general and might be safe to apply for new functionals, at least in combination with epc-17.2. Still, we will use the values in Table 2.

One further point we want to address is the robustness of the parametrization with respect to the basis set size. Therefore, we carried out additional one-dimensional Bayesian optimizations for NEO-B2PLYP and NEO-PBEQIDH utilizing different basis sets. The obtained results are shown in Figure S1 with corresponding optimization results in Table S1.

Both the def2-TZVPP/PB4-F2 and the larger combination of def2-QZVPP/PB6-F lead to comparable RMSDs. Across the different functionals, the errors of the obtained results are in good agreement with the smaller basis set combination, only the aug-cc-pVTZ-mc/PB4-F2 slightly increases the RMSD to 1.67 kcal mol^–1^ for NEO-B2PLYP and lowers the error of the NEO–PBEQIDH method to 1.40 kcal mol^–1^. The larger uncontracted basis sets (uncHq) agree in general with the original basis results with negligible shifts in the parametrization. Overall, these results show that the parametrization is fairly independent of the basis set combination when larger electronic basis sets are used. The def2-TZVPP basis set is somewhat limited and does require a slightly different value for the parameter *b*_epc_. We observe no significant changes in the performance or parametrization for basis sets larger than aug-cc-pVTZ-mc. It should be noted as well that although the larger basis set combinations achieve a similar performance on the RMSDs, the computational effort is drastically increased. The calculations with the optimized parameters for the CH_3_(CH_2_)_3_COO^–^ (**5**) molecule exhibits a 2.7 times higher CPU time for the aug-cc-pVTZ-mc/PB4-F2 basis set combination and a 5.2 times higher CPU time for the QZVPP/PB6-F basis set combination, compared to the CPU time of the def2-TZVPP/PB4-F2 basis set combination. Therefore, we recommend the usage of the proposed def2-TZVPP/PB4-F2 combination which offers an excellent trade-off between accuracy and computational effort.

### Performance of Multicomponent Double-Hybrid Functionals

#### Proton Affinity Test Set

In order to assess the performance of the parametrized multicomponent double-hybrid density functionals, we composed an additional test set for experimental proton affinities. Thereby, we follow the work of Moser et al.^78^ and consider proton affinities which are important in biocatalysis, a promising application area for multicomponent methods as recently demonstrated by Chow et al.^79^ The composition and the according experimental proton affinities of our test set are shown in Table 3. It should be highlighted that the test set is constructed by entirely different chemical motives (except one amine **7**′), providing an unbiased benchmark test set for our functionals. Moreover, the test set size is around 40% of the utilized PA21 data set size, yielding an overall 70:30 split between the data for parametrization and testing. The obtained UEs utilizing regular NEO–B3LYP and NEO–PBE0, as well as the NEO double-hybrid functionals for the test set are displayed in Figure 4. The first thing which becomes apparent is the outstanding performance of the NEO double-hybrid functionals in predicting proton affinities. The median UEs are all within or close to chemical accuracy with UEs of 0.86, 0.74, and 1.01 kcal mol^–1^ for the NEO-B2PLYP, NEO–DSD-PBEP86 and NEO-PBEQIDH functionals. In contrast, the regular NEO–DFT methods achieve median UEs of 3.03 and 3.16 kcal mol^–1^ for NEO–B3LYP and NEO–PBE0. The regular NEO–DFT methods show an evenly distributed range of unsigned errors, whereas the NEO–B3LYP distribution is narrower within 1.08–4.98 kcal mol^–1^. This can be compared to the wider error distribution of NEO–PBE0 ranging from 0.56 to 6.77 kcal mol^–1^. Contrary to the NEO–DFT counterparts, the NEO double-hybrid functionals exhibit a highly asymmetric distribution with half or more of the data points below 1 kcal mol^–1^. In general, the alcohol systems CH_3_CH_2_O^–^ (**1** ^**’**^) and (CH_3_)_2_CHO^–^ (**2** ^**’**^) are the most challenging for all tested functionals leading to the highest individual UEs above 3 kcal mol^–1^. Interestingly, there is one system where the regular NEO–DFT methods outperform the NEO double-hybrid functionals. In case of the p-C_6_H_4_NO_3_^–^ (**6** ^**′**^) molecule the regular NEO–DFT methods are providing estimates of the proton affinity which are around 1 kcal mol^–1^ lower in the UEs than the NEO double-hybrid methods. Regarding the results of the single-component based proton affinities on the NO_2_^–^ (**21**) molecule, it becomes apparent that the electronic structure of this chemical group in general is challenging, even more so if it is the protonation site. Overall, the benchmarked performance of the NEO double-hybrid functionals on the test set is remarkably accurate.

**Table 3:** Chemical Composition and Experimental Proton Affinities in kcal mol^–1^ of the Test Set^78^

index	Molecule	exp. PA
alcohols		
**1** ^**′**^	CH_3_CH_2_O^–^	378.2
**2** ^**′**^	(CH_3_)_2_CHO^–^	375.7
phenols		
**3** ^**′**^	o-C_6_H_4_ClO^–^	343.4
**4** ^**′**^	m-C_6_H_4_ClO^–^	342.1
**5** ^**′**^	p-CH_3_C_6_H_4_O^–^	350.7
**6** ^**′**^	p-C_6_H_4_NO_3_^–^	327.8
amine and N-heterocycles		
**7** ^**′**^	(CH_3_)_2_CHCH_2_NH_2_	220.8
**8** ^**′**^	C_3_H_4_N_2_	225.3
**9** ^**′**^	(CH_2_)_4_NH	226.6

**Figure 4:**
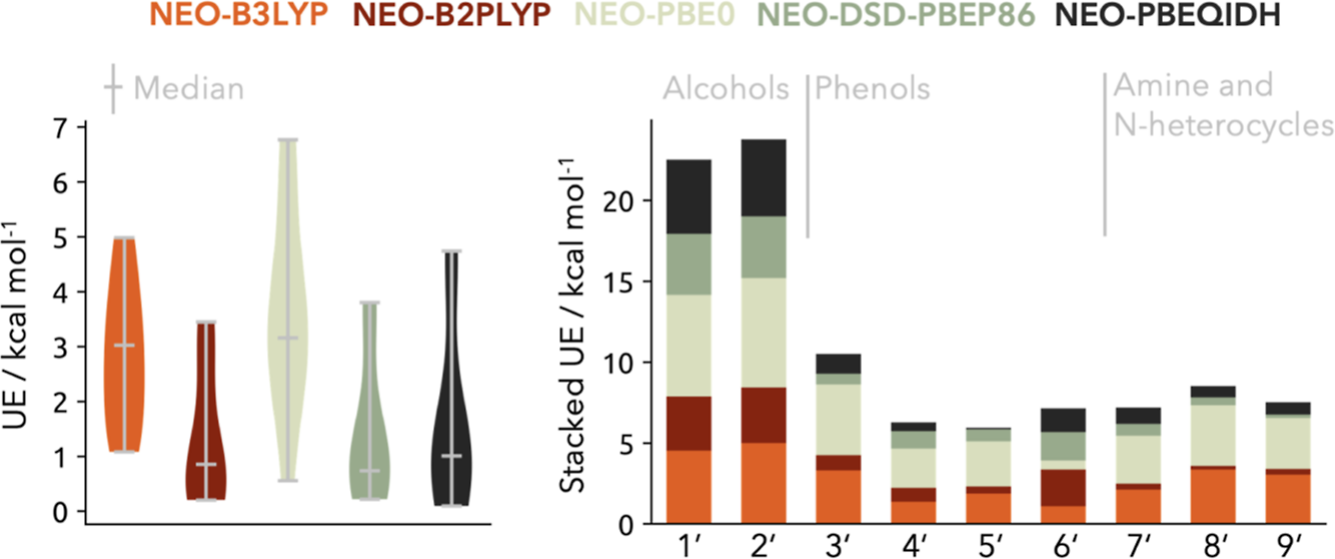
Left: Violin plots with highlighted median of the overall performance of the multicomponent methods given as unsigned error on the test data set. Right: Stacked unsigned errors of the respective methods for each molecule in the test data set.

#### Protonated Water Hexamers

The next benchmark systems we would like to introduce are the isomers of the protonated water hexamer. The isomers of the protonated water tetramers were already investigated in multiple studies and exemplify the outstanding benefit of directly including anharmonic zero-point vibrational energies within multicomponent methods.^51,52,76,80^ The larger protonated water hexamers have received less attention so far. Fetherolf et al.^76^ carried out an in-depth study on the energetic ordering of the protonated water hexamer isomers based on single- and multicomponent Møller–Plesset second-order perturbation theory. The effects of orbital optimization, spin-scaling as well as the impact of harmonic and anharmonic zero-point vibrational energies were also analyzed. The conclusion of their study is that anharmonicity plays a crucial role in determining their relative energies. The harmonic approximation overestimates the impact of zero-point vibrational energy.^76^ Within this study we explore if the use of density functional theory will result in the same outcome. Therefore, we computed the energetic ordering with single-component methods, namely B3LYP and B2PLYP, employing harmonic zero-point vibrational energies and compare these to multicomponent NEO–B3LYP and NEO–B2PLYP energies, inherently including anharmonic zero-point vibrational energies of the quantum protons. The obtained orderings are shown in Figure 5. In general, the contribution of the oxygen atoms to the zero-point vibrational energy is negligible as demonstrated by Fetherolf et al.^76^

**Figure 5:**
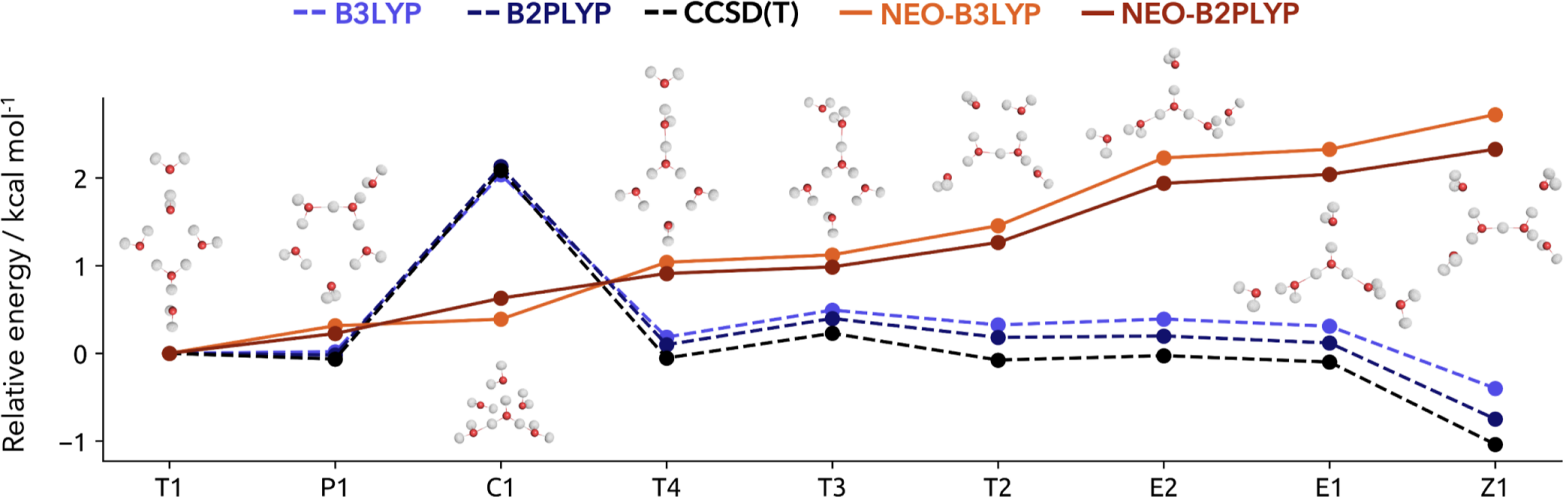
Relative energies of the protonated water hexamer isomers obtained with single-component based (dashed blue tones), multicomponent (solid red tones) hybrid and double-hybrid density functional theory, and conventional CCSD­(T) (dashed black) with respect to the T1 isomer.

We first discuss the different energetic ordering between the single- and multicomponent methods before we compare the influence of the hybrid and double-hybrid energies. The structures are ordered following the best available anharmonic estimates.^76^ The inclusion of harmonic and anharmonic zero-point vibrational energies leads to entirely different energetic orderings of the protonated water hexamer isomers. The multicomponent methods favor energetically the isomers with 6 hydrogen bonds present in the cluster, while the clusters with 5 hydrogen bonds are energetically the least favored. However, the C1 isomer features 8 hydrogen bonds within the cluster. This isomer exhibits two very strong hydrogen bonds with a bond length of 2.5 Å, one 2.6 Å hydrogen bond, three 2.8 Å hydrogen bonds and two 2.9 Å hydrogen bonds. Hereby, three water molecules are shared acceptors for two hydrogen bonds. Moreover, the compact geometry leads to suboptimal angles for hydrogen bonds. The energetically most stable isomer T1 features two almost shared protons with 2.5 Å hydrogen bond lengths, one 2.6 Å hydrogen bond, one 2.7 Å hydrogen bond and two 2.8 Å hydrogen bonds. Only one water molecule is a shared acceptor for two hydrogen bonds. In contrast, the less favorable C1 structure exhibits more hydrogen bond interactions compared to T1, which has stronger but fewer hydrogen bond interactions. In case of the single-component based results with harmonic zero-point vibrational energies, the lack of anharmonicity results in C1 being the least stable structure. Overall, the Z1 isomer with fewer hydrogen bond interactions is the most stable in the harmonic single-component picture, whereas the opposite result is achieved by inclusion of the anharmonicity via the multicomponent methods. In general, the energetic differences of the T, P, and E type structures are too small within the harmonic approximation. Additionally, for both the harmonic and multicomponent approximation, the difference between regular hybrid and double-hybrid density functional is very small. Nonetheless, the effect is not perfectly additive. Compared to the CCSD­(T) results, the single-component DFT methods yield orderings that closely match those obtained when higher-level electronic correlation effects are included. Overall, multicomponent double-hybrids offer an accurate and computational feasible alternative to include anharmonic zero-point vibrational effects, which are important to archive the correct energetic ordering of large water clusters.^76^ Moreover, we could demonstrate the robustness of our parametrization which largely inherited the features of the employed single-component based double-hybrid density functionals.

#### Protonated 12-Crown-4 Ether

Following the prior benchmarking we depict one further challenging example for single-component methods, which is the protonated 12-crown-4 ether. Recently, Torres-Boy et al.^81^ investigated the proton bonding of the protonated 12-crown-4 ether in liquid helium droplets at sub-Kelvin temperatures via infrared laser spectroscopy. Thereby, creating an ideal environment to elucidate the proton dynamics free from thermal fluctuations of the molecular cage. They observed narrow vibrational bands, hinting toward a robust proton bond bridging both ether sites. The corresponding energy surface of the proton bond is wide and highly anharmonic. As a result of this, single-component harmonic calculations fail to describe the experimental band positions. Moreover, employing perturbative anharmonic corrections is costly and does not give a full picture of how bonding is impacted by these effects.

We have computed the relative energies of this system with the regular single- and multicomponent B2PLYP double-hybrid density functional for both bonding motives, where the proton is located on one site or shared between the oxygen atoms. The results are shown in Figure 6. The two methods deliver substantially different results. The single-component picture is favoring the localization of the proton to one of the oxygen sites by virtue of a double-well potential with a barrier of 3.0 kJ mol^–1^. The multicomponent approach produces a shared proton. This should impact the geometry and dynamics of the system, allowing for a better understanding of the experimental results. However, we are still to develop gradients for our hybrid approach.

**Figure 6:**
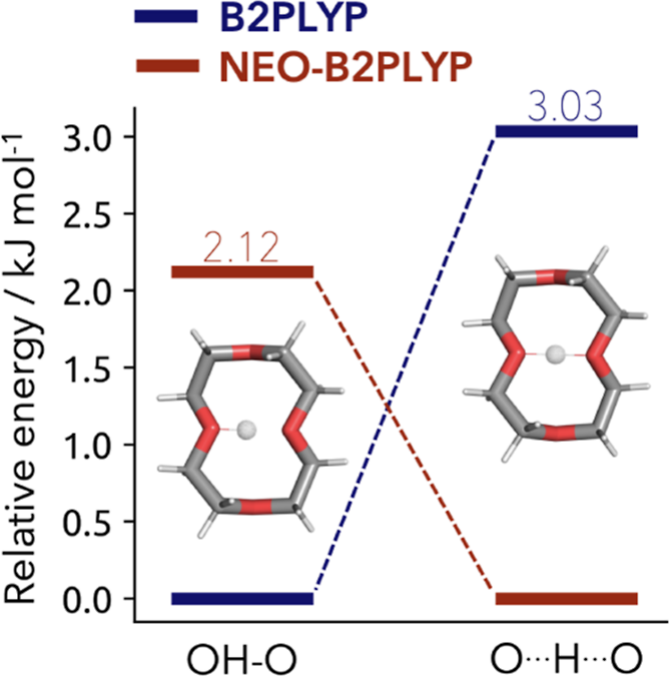
Relative energies of the 12-crown-4 ether with the proton located on one site (left) and a shared proton (right) obtained with single- (blue) and multicomponent (red) B2PLYP.

With the aim to obtain vibrational frequencies, which can be compared to the experimental bands, NEO dynamics can be carried out utilizing the NEO double-hybrid functionals. The Fourier transform of the obtained dipole moment autocorrelation function can be employed to compute the respective frequencies, as successfully demonstrated by Zhang et al.^82^ for hybrid constrained NEO–DFT functionals. For the protonated 12-crown-4 ether system the increase in the computational effort by the additional density fitted NEO–MP2 calculation is only 1% compared to the underlying NEO–DFT computation. This is due to the large number of iterations required in the NEO SCF procedure. The MP2 correction is obtained in one-shot with a very low prefactor.

## Conclusions

In this work we explore for the first time the admixture of NEO–DFT and NEO–MP2 electron–proton correlation energies. Three double-hybrid functionals (B2PLYP, DSD–PBEP86 and PBEQIDH) are parametrized by making use of proton affinities from the PA23 data set. The parameter space was investigated with a Bayesian optimization algorithm. The parametrization of all three models show that values around a 0.8:0.2 fraction in NEO–DFT and NEO–MP2 electron–proton correlation leads to a visible improvement in the accuracy, resulting in a halved RMSD. This has been confirmed for an independent set of proton affinities, which served as test set. The parameters for each model are provided to facilitate future applications and further development. Test calculations with varying basis set sizes shows that the parametrization is quite robust. We have also applied the model to the energetics of protonated water hexamers and the calculation of the relative stability of crown-ether molecular isomers.

It should be noted that for the first time one observes the use of multicomponent MP2 (without orbital optimization) for quantitative descriptions of chemical phenomena. NEO–MP2 is known to do little in correcting for the shortcomings of NEO–HF. This is connected to the overlocalized NEO–HF protonic orbitals, which require relaxation effects to be included in correlated calculations. In the case of double-hybrid functionals as proposed in this work, the starting orbitals are from NEO–DFT and already include some level of relaxation. We believe this to be one of the main reasons why MP2 is able to perform beyond what has been so far documented. It should be noted though, that even our results advise only a rather modest admixing of MP2 correlation (about 20%).

One of the difficulties faced in this work was the lack of reference data for parameter optimization. If one looks at the standard for electronic structure models, the amount of data used in this work is subpar. It is reassuring to observe, however, that the DFT/MP2 ratio we obtained is still robust to several different variations. We are currently in the process of gathering reference proton energies and densities in model systems, data that can be used to further confirm the parametrization. Spectroscopic data could also be helpful in that respect.^32,83,84^ Our hope is that with the added interest in multicomponent methods the benchmarking efforts will be intensified over the next years, providing a more solid base for (meta-)­GGA and double-hybrid functionals.

## Supplementary Material



## Data Availability

Structural information are available free of charge on GRO.data (DOI: 10.25625/XKTMTI).

## Notes

Bayesian optimizations for NEO–B2PLYP and NEO–PBEQIDH utilizing different basis sets Figure S1, with corresponding optimization results in Table S1, specific formulation of the multicomponent double-hybrid functionals (PDF)
