# Supplementary material for: Multicomponent Double-Hybrid Density Functional Theory
Source: J Chem Theory Comput. 2025 Nov 7;21(22):11509–20. doi: 10.1021/acs.jctc.5c01222 (PMC12674594; doi:10.1021/acs.jctc.5c01222)
Supplement: Supplementary file 1 [file ct5c01222_si_001.pdf]

Supporting Information: Multicomponent Double-Hybrid Density Functional Theory

Lukas Hasecke* and Ricardo A. Mata*

Institute of Physical Chemistry, University of Göttingen, Tammannstrasse 6, 37077, Göttingen, Germany

E-mail: lhaseck@gwdg.de; rmata@gwdg.de

This supporting information contains additional data and analysis related to the Bayesian optimization of electron-proton correlation parameters, as well as the full formulation of the developed multicomponent double-hybrid functionals. Specifically, Table S1 presents the optimized values of b_{epc} (epc-17.2) and c_{epc} (MP2) for various basis set combinations, along with the corresponding RMSDs in kcal mol^{-1}. Figure S1 illustrates the Bayesian optimization procedure on the PA21 data set, showing the RMSD achieved for each tested parameter set and basis set combination. The last page provides the specific formulation of the double-hybrid functionals. One should note that for the purely electronic terms default Molpro library values have been applied (as in the single-component formulation).

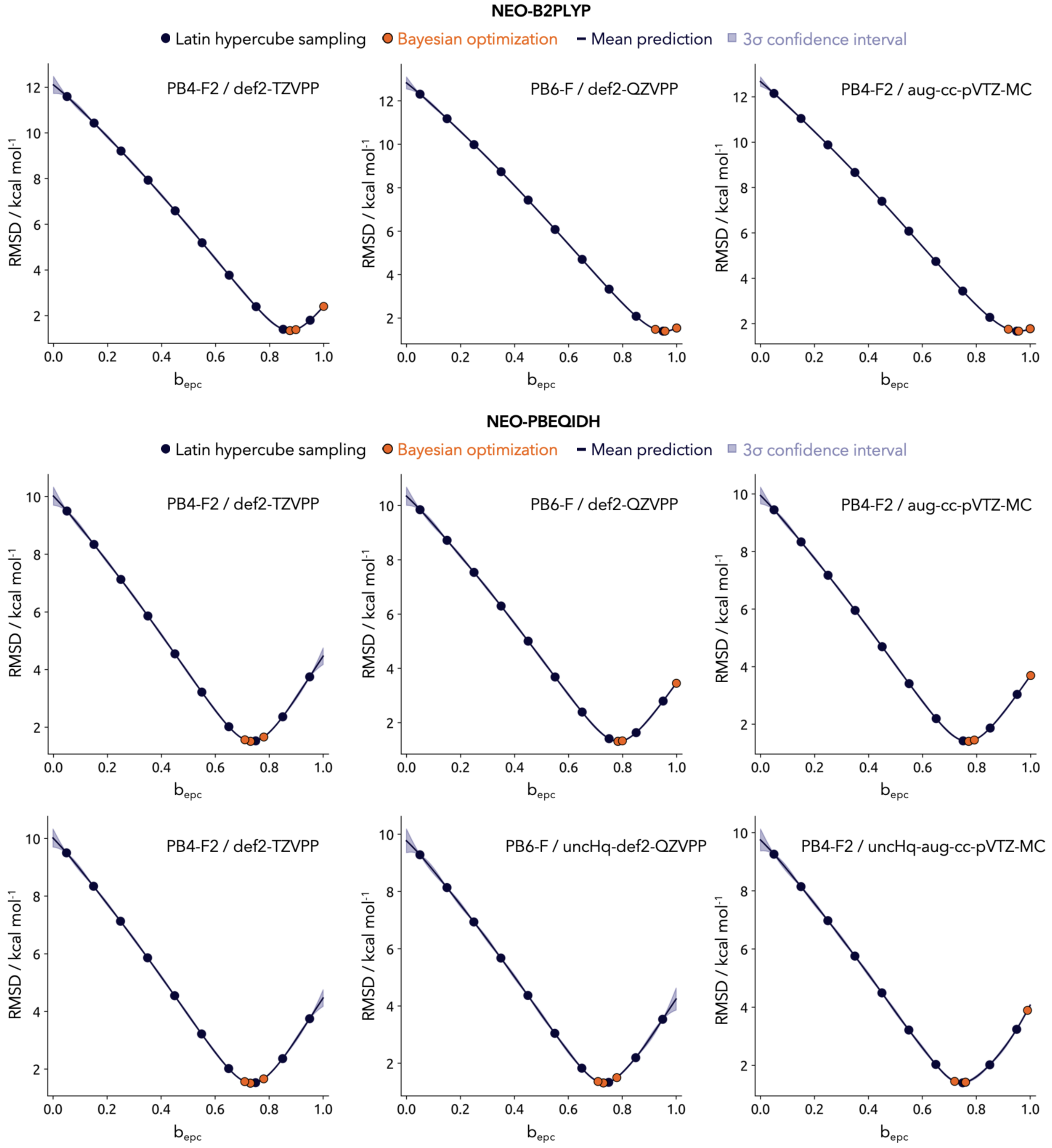

Figure S 1: Bayesian optimization of the parameters for the admixture of electron-proton correlation obtained with the epc-17.2 b_{epc} and MP2 $c_{\mathrm{epc}} = 1 - b_{\mathrm{epc}}$ on the PA21 data set of proton affinities shown with the achieved RMSD for the respective double-hybrid density functionals for various basis set combinations.

Table S 1: Obtained parameters for the admixture of epc-17.2 (b_{epc}) and MP2 (c_{epc}) electron-proton correlation based on the Bayesian optimization employing the basis set combinations shown in Fig. S1 with resulting RMSDs in kcal mol^{-1}.

Functional	b_{epc}	c_{epc}	RMSD
		def2-TZVPP/PB4-F2	
NEO-B2PLYP	0.875	0.125	1.34
NEO-PBEQIDH	0.731	0.269	1.51
		def2-QZVPP/PB6-F	
NEO-B2PLYP	0.957	0.043	1.39
NEO-PBEQIDH	0.782	0.218	1.31
		aug-cc-pVTZ-MC/PB4-F2	
NEO-B2PLYP	0.957	0.043	1.67
NEO-PBEQIDH	0.771	0.229	1.40
		uncHq-def2-QZVPP/PB6-F	
NEO-PBEQIDH	0.731	0.269	1.31
		uncHq-aug-cc-pVTZ-MC/PB4-F2	
NEO-PBEQIDH	0.750	0.250	1.40

NEO-B2PLYP

$$
\begin{aligned}
E_{\mathrm{XC}} =& 0.47 \cdot E_{\mathrm{X}}(\mathrm{B88}) + 0.53 \cdot E_{\mathrm{X}}(\mathrm{HF}) + 0.73 \cdot E_{\mathrm{C}}(\mathrm{LYP}) + 0.27 \cdot E_{\mathrm{C}}(\mathrm{MP2}) \\
&+ 0.875 \cdot E_{\mathrm{EPC}}(\mathrm{EPC\text{-}17.2}) + 0.125 \cdot E_{\mathrm{EPC}}(\mathrm{MP2})
\end{aligned}
$$

NEO-DSD-PBEP86

$$
\begin{aligned}
E_{\mathrm{XC}} =& 0.31 \cdot E_{\mathrm{X}}(\mathrm{PBE}) + 0.69 \cdot E_{\mathrm{X}}(\mathrm{HF}) + 0.44 \cdot E_{\mathrm{C}}(\mathrm{P86}) + 0.22 \cdot E_{\mathrm{C}}(\mathrm{MP2\text{-}SS}) + 0.52 \cdot E_{\mathrm{C}}(\mathrm{MP2\text{-}OS}) \\
&+ 0.806 \cdot E_{\mathrm{EPC}}(\mathrm{EPC\text{-}17.2}) + 0.194 \cdot E_{\mathrm{EPC}}(\mathrm{MP2})
\end{aligned}
$$

NEO-PBEQIDH

$$
\begin{aligned}
E_{\mathrm{XC}} =& 0.3066 \cdot E_{\mathrm{X}}(\mathrm{PBE}) + 0.6934 \cdot E_{\mathrm{X}}(\mathrm{HF}) + 0.6667 \cdot E_{\mathrm{C}}(\mathrm{PBE}) + 0.3333 \cdot E_{\mathrm{C}}(\mathrm{MP2}) \\
&+ 0.731 \cdot E_{\mathrm{EPC}}(\mathrm{EPC\text{-}17.2}) + 0.269 \cdot E_{\mathrm{EPC}}(\mathrm{MP2})
\end{aligned}
$$
